# Supplementary material for: The usherin mutation c.2299delG leads to its mislocalization and disrupts interactions with whirlin and VLGR1
Source: Nat Commun. 2023 Feb 21;14:972. doi: 10.1038/s41467-023-36431-1 (PMC9944904; doi:10.1038/s41467-023-36431-1)
Supplement: Supplementary file 1 — Supplementary Information [file 41467_2023_36431_MOESM1_ESM.pdf]

Supplementary Information for

The usherin mutation c.2299delG leads to its mislocalization and disrupts interactions with whirlin and VLGR1

Lars Tebbe[1,#], Maggie L. Mwoyosvi[1,3,#], Ryan Crane[1], Mustafa S. Makia[1], Mashal Kakakhel[1], Dominic Cosgrove[2], Muayyad R. Al-Ubaidi[1,*], and Muna I. Naash[1,*]

[1]Department of Biomedical Engineering, University of Houston, Houston TX, 77204, USA

[2]Boys Town National Research Hospital, Omaha, Nebraska, USA

[3]Current address: Department of Microbiology & Immunology, University of Oklahoma Health Sciences Center, Oklahoma City, OK 73104, USA

Classification: Biological Sciences, Cell Biology

[#]These authors made equal contributions

*To whom correspondence should be addressed at: Muna I. Naash (ORCID: 0000-0002-6534-5144), mnaash@central.uh.edu; Phone: 713-743-1651 (MIN) and Muayyad R. Al-Ubaidi (ORCID: 0000-0002-4914-350X), malubaid@central.uh.edu; Phone: 713-743-1648 (MRA); Department of Biomedical Engineering, University of Houston, 3517 Cullen Blvd. Room 2027, Houston, TX 77204-5060.

Supplementary Table 1. List of antibodies used in the study and their sources.

Antigen	Species	Application/Concentration	Source	RRID#
Acetylated tubulin	Mouse	1:400 (IF)	Sigma-Aldrich, (T7451)	AB_609894
Actin (HRP-	Mouse	1:20000 (IB)	Sigma-Aldrich, (A3854)	AB_262011
Calreticulin	Chicken	1:200 (IF)	Invitrogen, (PA1-902A)	AB_2069607
CDH23	Rabbit	1:50 (IF)	Dr. Dominic Cosgrove[1]	NA
Cen2	Rabbit	1:1000 (IF)	Dr. Uwe Wolfrum[2]	NA
CIB2	Mouse	1:200 (IF)	Abnova, (H00010518-A01)	AB_489656
Clarin 1	Rabbit	1:700 (IF)	Dr. Dominic Cosgrove[3]	NA
FLAG	Rabbit	1:1000 (IB)	Cell Signaling, (14793)	AB_2572291
FLAG M2	Mouse	1:100 (IF); 1:1000 (IB)	Sigma-Aldrich, (F1804)	AB_262044
FLAG	Rat	1:100 (IF)	Biolegends (637301)	AB_1134266
GAPDH	Chicken	1:1000 (IB)	Sigma-Aldrich, (AB2302)	AB_10615768
GAPDH	Mouse	1:1000 (IB)	Proteintech, (60004-1-Ig)	AB_2107436
GFAP	Mouse	1:500 (IF)	Chemicon, (MAB360)	AB_11212597
GFP	Rabbit	1:1000 (IB, IF)	Invitrogen, (A11122)	AB_221569
Harmonin	Rabbit	1:25 (IF)	Dr. Dominic Cosgrove[4]	NA
Human-20 amino acid	Rabbit	1:500 (IF)	Century Biochemicals,	NA
IRBP	Rabbit	1:1000 (IB)	Dr. Gregory Liou[5]	AB_2631101
MWL-Opsin 1	Rabbit	1:5000 (IF)	Novus, (NB110-74730)	AB_1049390
Opsin (bovine)	Rabbit	1:2000 (IF)	Dr. Steven J. Fliesler[6]	NA
PCDH15	Rabbit	1:150 (IF)	Dr. Dominic Cosgrove[1]	NA

Prph2	Rabbit	1:1000 (IB)	In house[7]	AB_2833006
SANS	Rabbit	1:25 (IF)	Dr. Dominic Cosgrove[4]	NA
S-Opsin	Goat	1:500 (IF)	Santa Cruz Biotech, (sc-14363)	AB_2158332
Stx3	Rabbit	1:500 (IF)	Novus, (NBP1-86984)	AB_11055708
Usherin b	Rabbit	1:5000 (IF, IB)	Dr. Jun Yang[8]	NA
Whrn	Rabbit	1:100 (IF)	Proteintech, (25881-1-AP)	AB_2880280
Vlgr1	Rabbit	1:250 (IF)	Dr. Uwe Wolfrum[9]	NA
Vlgr1	Chicken	1:2000 (IF)	Dr. Jun Yang[8]	NA

Supplementary Table 2. List of primers used in the study.

**Target**	**Primer**	**Sequence**
total message	forward primer	5’-CCGAATTGACCTTAGCTGTGTGGTTG-3’
total message	reverse primer	5’-GTCCTACTTGTATCACGCTGGAGAC-3’
Truncated mutant message	forward primer	5’-GGGACCGTGGATGGAGACATTAC-3’
Truncated mutant message	reverse primer	5’-GGTGTCACACTGAAGTCCTTTGGC-3’
WT message:	forward primer	5’-GGGACCGTGGATGGAGACATTAC-3’
WT message:	reverse primer	5’-CACTGCCCTGTCTCAGTGTCACA-3’

Supplementary Figures

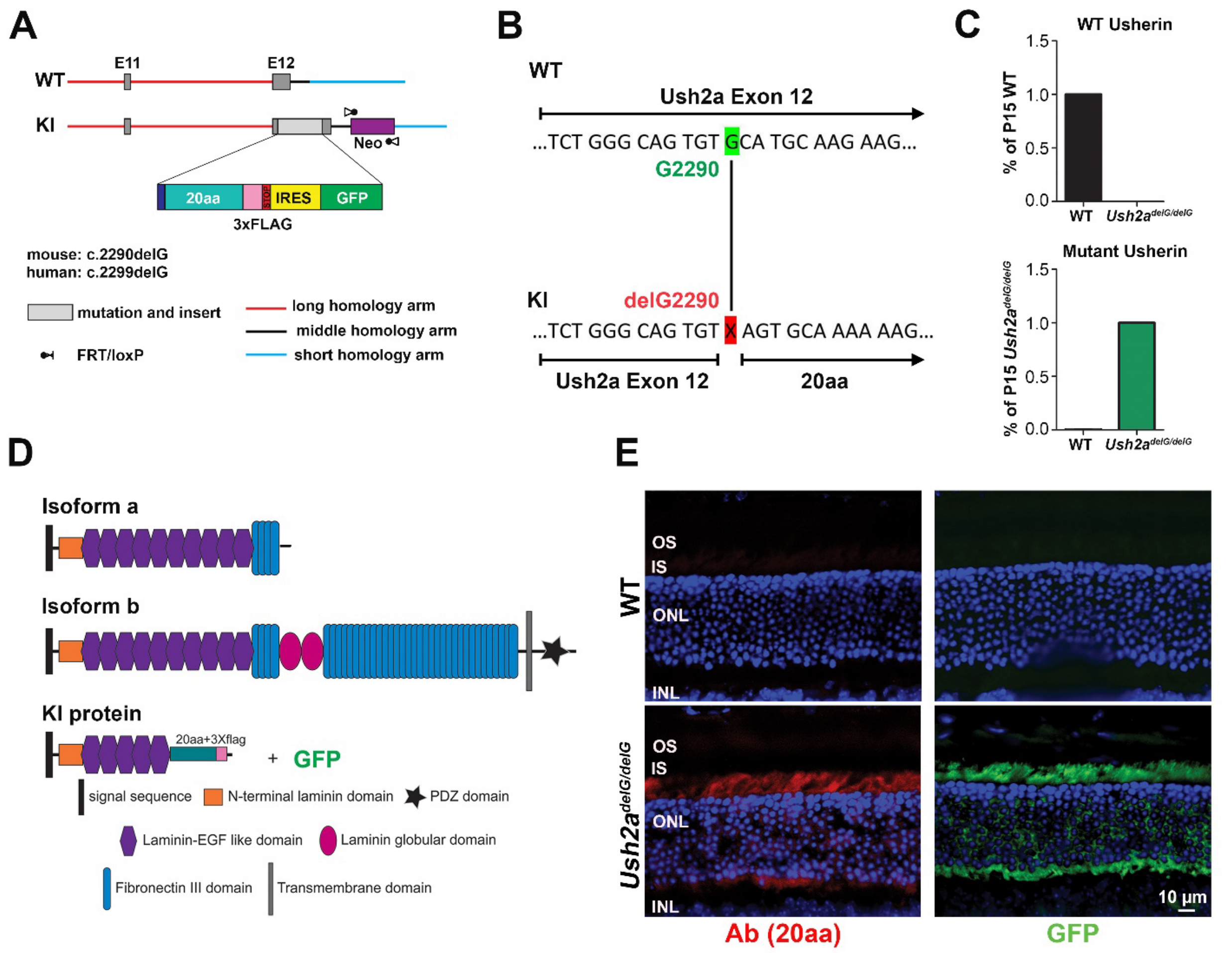

Supplementary Figure 1. Design strategy for the KI model. (**A**) Schematic representation of the USH2A c.2299delG targeted KI strategy. (**B**) Sequence comparison between WT and KI at the site of the mutation. (**C**) WT transcript is present in P15 WT and absent in P15 KI retinas while mutant transcript is expressed in KI but not in WT retinas. Samples were normalized to the expression levels of HPRT. Shown are mean ± SEM from four independent samples. (**D**) Diagram depicting the functional domains of the proteins expressed by WT and KI mice. (**E**) Retinal sections from P30 WT and *Ush2a$^{delG/delG}$* with antibodies against the human 20 amino acids extension (left images) and GFP (right images). These results were reproduced in three independent experiments. Single plane images were captured showing the presence of mutant usherin and GFP only in the *Ush2a$^{delG/delG}$* retina. OS: outer segment; IS: inner segment; ONL: outer nuclear layer; INL: inner nuclear layer.

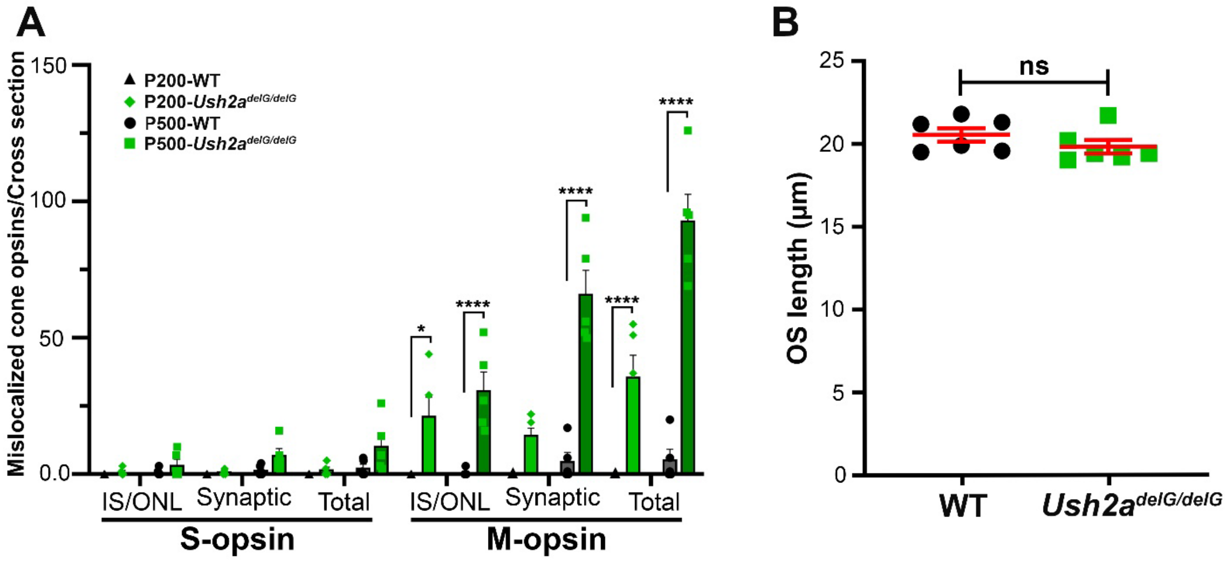

Supplementary Figure 2. Mislocalization of cone opsins. (**A**) Total number of S- versus M-opsin mislocalized to the IS/ONL and synaptic terminals of P200 and P500 WT and *Ush2a*$^{delG/delG}$ mice. Cones with mislocalized opsin were counted from entire retinal cross sections shown in Fig. 4D. N-values: WT: P200: 4; P500: 5. *Ush2a*$^{delG/delG}$: P200: 5; P500: 5. Data is shown as mean ± SEM. Significance was determined by a two-way ANOVA with a Boferroni's post hoc test. P-values: M-Opsin: IS/ONL: P200: p=0.0330 (*), P500: p<0.0001 (****). Synaptic: P500: p<0.0001 (****). Total: P200: p<0.0001 (****), P500: p<0.0001 (****). (**B**) Length of the OS in the WT and *Ush2a*$^{delG/delG}$ retinas. Data is shown as mean ± SEM. No significant change in the length of the OS could be observed in the *Ush2a*$^{delG/delG}$ retina. Measurements were obtained from an image inferior and another superior and performed on three independent mice for each genotype. Lack of significance was determined by a two-tailed unpaired t-test.

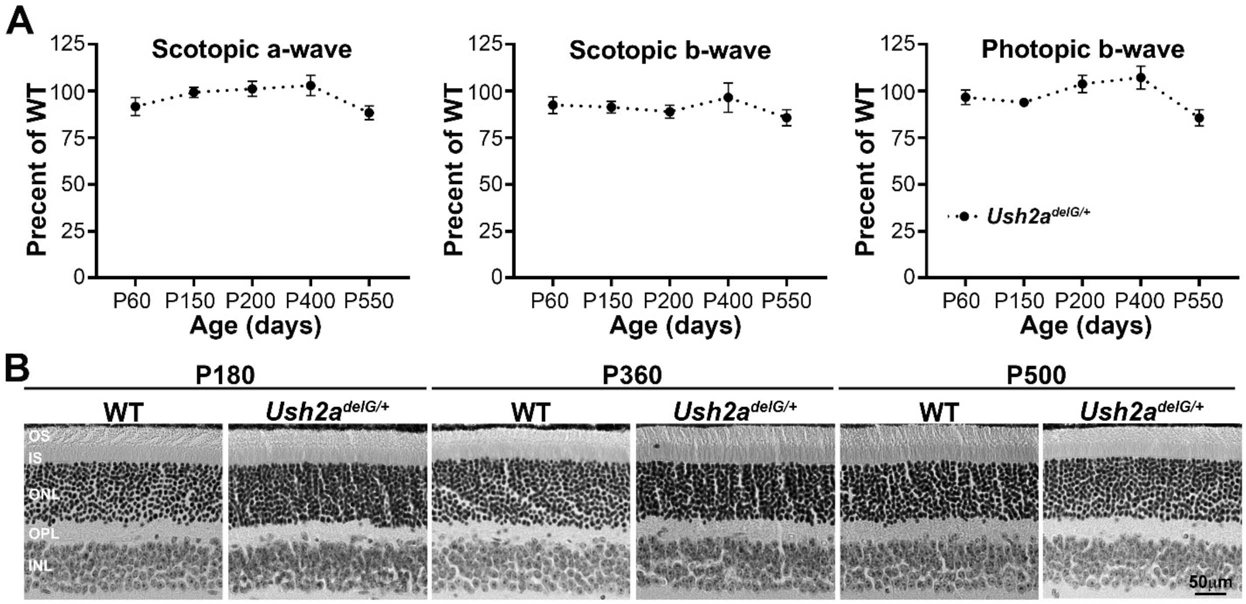

Supplementary Figure 3. ***Ush2a***$^{delG/+}$ **mice display no loss in photoreceptor function.** (**A**) Full-field scotopic and photopic ERGs were performed on WT and *Ush2a*$^{delG/+}$ mice at the indicated ages. No significant reduction was observed at any time point measured for both of scotopic a- and b-wave, or for photopic b-wave. Significance test by two-way ANOVA with Bonferroni's post hoc comparison. Shown are mean ±SEM. N-values: Scotopic a-wave: P60: 11; P150: 14; P200: 15; P400: 12; P500: 10. Scotopic b-wave: P60: 11; P150: 14; P200: 14; P400: 8; P500: 11. Photopic b-wave: P60: 11; P150: 7; P200: 13; P400: 11; P500: 10. Same amount of mice were used for both WT and *Ush2a*$^{delG/+}$. (**B**) Histological assessments of the WT and *Ush2a*$^{delG/+}$ retinas at the indicated ages showing no gross structural differences were noted from the age-matched controls.

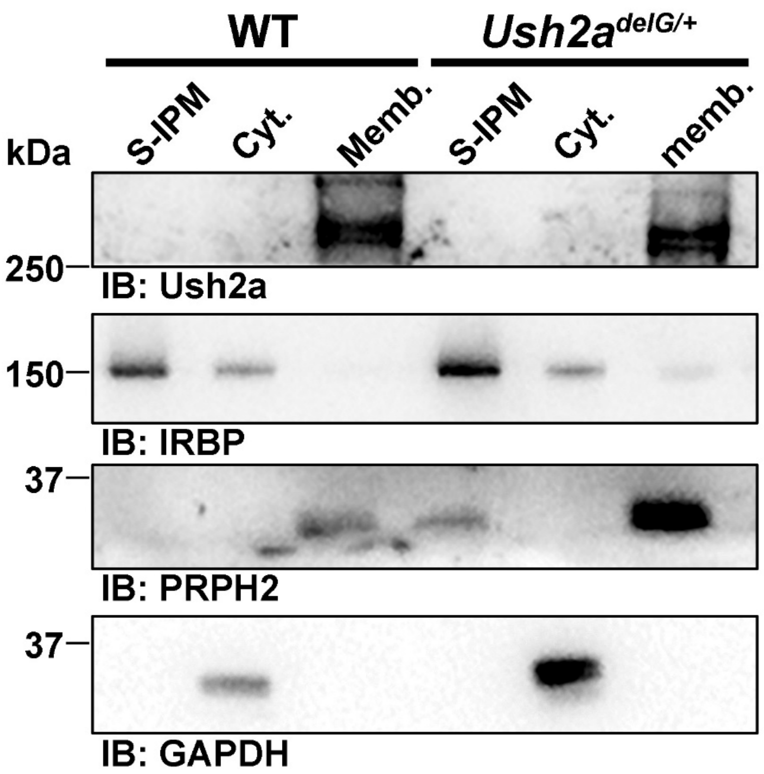

Supplementary Figure 4. Fractionation of WT and *Ush2a*$^{delG/+}$ retinal extracts. The fractionation shows that full length usherin is exclusively localized in the membrane bound fraction of retinal extracts taken from P30 WT and *Ush2a*$^{delG/+}$ retina. GAPDH was used as cytosolic marker, IRBP as an S-IPM marker, while PRPH2 was used to mark the membrane fraction. The same results were obtained in three independent experiments. S-IPM: soluble inter photoreceptor matrix, Cyt.: cytosolic; Memb.: membrane.

References:

1 Zallocchi, M., Sisson, J. H. & Cosgrove, D. Biochemical characterization of native Usher protein complexes from a vesicular subfraction of tracheal epithelial cells. *Biochemistry* **49**, 1236-1247, (2010).

2 Giessl, A. *et al.* Differential expression and interaction with the visual G-protein transducin of centrin isoforms in mammalian photoreceptor cells. *The Journal of biological chemistry* **279**, 51472-51481, (2004).

3 Zallocchi, M. *et al.* Localization and expression of clarin-1, the Clrn1 gene product, in auditory hair cells and photoreceptors. *Hearing research* **255**, 109-120, (2009).

4 Zallocchi, M. *et al.* EIAV-based retinal gene therapy in the shaker1 mouse model for usher syndrome type 1B: development of UshStat. *PloS one* **9**, e94272, (2014).

5 Schneider, B. G., Papermaster, D. S., Liou, G. I., Fong, S. L. & Bridges, C. D. Electron microscopic immunocytochemistry of interstitial retinol-binding protein in vertebrate retinas. *Invest Ophthalmol Vis Sci* **27**, 679-688, (1986).

6 Tan, E. *et al.* The relationship between opsin overexpression and photoreceptor degeneration. *Invest Ophthalmol Vis Sci* **42**, 589-600, (2001).

7 Ding, X. Q., Stricker, H. M. & Naash, M. I. Role of the second intradiscal loop of peripherin/rds in homo and hetero associations. *Biochemistry* **44**, 4897-4904, (2005).

8 Zou, J. *et al.* Individual USH2 proteins make distinct contributions to the ankle link complex during development of the mouse cochlear stereociliary bundle. *Hum Mol Genet* **24**, 6944-6957, (2015).

9 Reiners, J. *et al.* Scaffold protein harmonin (USH1C) provides molecular links between Usher syndrome type 1 and type 2. *Hum Mol Genet* **14**, 3933-3943, (2005).
