# Supplementary material for: The usherin mutation c.2299delG leads to its mislocalization and disrupts interactions with whirlin and VLGR1
Source: Nat Commun. 2023 Feb 21;14:972. doi: 10.1038/s41467-023-36431-1 (PMC9944904; doi:10.1038/s41467-023-36431-1)
Supplement: Supplementary file 3 — Reporting Summary [file 41467_2023_36431_MOESM3_ESM.pdf]

nature portfolio

Corresponding author(s): Muna Naash

Last updated by author(s): Jan 19, 2023

Reporting Summary

Nature Portfolio wishes to improve the reproducibility of the work that we publish. This form provides structure for consistency and transparency in reporting. For further information on Nature Portfolio policies, see our Editorial Policies and the Editorial Policy Checklist.

Statistics

For all statistical analyses, confirm that the following items are present in the figure legend, table legend, main text, or Methods section.

n/a	Confirmed	
☐	☒	The exact sample size (n) for each experimental group/condition, given as a discrete number and unit of measurement
☐	☒	A statement on whether measurements were taken from distinct samples or whether the same sample was measured repeatedly
☐	☒	The statistical test(s) used AND whether they are one- or two-sided<br>*Only common tests should be described solely by name; describe more complex techniques in the Methods section.*
☐	☒	A description of all covariates tested
☐	☒	A description of any assumptions or corrections, such as tests of normality and adjustment for multiple comparisons
☐	☒	A full description of the statistical parameters including central tendency (e.g. means) or other basic estimates (e.g. regression coefficient) AND variation (e.g. standard deviation) or associated estimates of uncertainty (e.g. confidence intervals)
☐	☒	For null hypothesis testing, the test statistic (e.g. F, t, r) with confidence intervals, effect sizes, degrees of freedom and P value noted<br>*Give P values as exact values whenever suitable.*
☒	☐	For Bayesian analysis, information on the choice of priors and Markov chain Monte Carlo settings
☒	☐	For hierarchical and complex designs, identification of the appropriate level for tests and full reporting of outcomes
☒	☐	Estimates of effect sizes (e.g. Cohen's d, Pearson's r), indicating how they were calculated

Our web collection on statistics for biologists contains articles on many of the points above.

Software and code

Policy information about availability of computer code

Data collection	ERG: EMwin, Version 9.8.0, LKC technologies<br>Microscopy, IHC: Zen 2.3, Version 2.3.64.0, Zeiss GmbH<br>Microscopy, Histology: Zen 2, Version 2.0.0.0, Zeiss GmbH<br>Immuno blots: ImageLab 6.1, Version 6.1.0 build 7, BioRad Laboratories<br>qRT-PCR: CFX Manager, Version 2.1.1022.0523, BioRad Laboratories
Data analysis	ERG: EMwin, Version 9.8.0, LKC technologies<br>Ciliary length measurement IHC, OS and CC width measurements EM: ImageJ, Version 1.53f51, NIH<br>Immuno blots: ImageLab 6.1, Version 6.1.0 build 7, BioRad Laboratories<br>qRT-PCR: CFX Manager, Version 2.1.1022.0523, BioRad Laboratories<br>qRT-PCR, sorting of dataset: Excel 2016, Version 16.0.5378.1000, Microscoft<br>Graphs and statistics: Prism 7, Version 7.05, GraphPad Software, Inc.<br>Assembling of Scheme: BioRender, biorender.com<br>Assembling of figures: Photoshop 2020, Version 21.0.3<br>OS length measurements: Zen 3.5, Version 3.5.093.00001

For manuscripts utilizing custom algorithms or software that are central to the research but not yet described in published literature, software must be made available to editors and reviewers. We strongly encourage code deposition in a community repository (e.g. GitHub). See the Nature Portfolio guidelines for submitting code & software for further information.

Data

Policy information about availability of data

All manuscripts must include a data availability statement. This statement should provide the following information, where applicable:

- Accession codes, unique identifiers, or web links for publicly available datasets
- A description of any restrictions on data availability
- For clinical datasets or third party data, please ensure that the statement adheres to our policy

All data generated or analyzed during this study are included in this article and its supplementary information files.

Human research participants

Policy information about studies involving human research participants and Sex and Gender in Research.

Reporting on sex and gender	No human subjects were used
Population characteristics	NA
Recruitment	NA
Ethics oversight	NA

Note that full information on the approval of the study protocol must also be provided in the manuscript.

Field-specific reporting

Please select the one below that is the best fit for your research. If you are not sure, read the appropriate sections before making your selection.

☒ Life sciences ☐ Behavioural & social sciences ☐ Ecological, evolutionary & environmental sciences

For a reference copy of the document with all sections, see nature.com/documents/nr-reporting-summary-flat.pdf

Life sciences study design

All studies must disclose on these points even when the disclosure is negative.

Sample size	The numbers of each individual experiment are stated in the manuscript. For qualitative experiments a sample size of at least three (repeated on at least three separate occasions) was chosen based on our prior work. In qualitative experiments, the changes we describe are visually evident (e.g. a band shift on a western blot in deglycosylation experiments) and were repeatable through different replicates. For quantitative experiments, we did not perform sample size/power analysis calculations prior to the start of the study. Sample sizes were chosen based on our past experience with inbred, genetically modified models of retinal degeneration. Sample sizes are typically larger for physiological and functional measurements (such as ERG) where our past experience has shown that there is more inter-animal variability. In past functional studies using our models of inherited retinal degeneration, sample sizes in the range of 5-10 animals per group have been sufficient to detect (with statistical significance) differences between means on the order of ~20-25%, so we use that as a starting target goal for each sample size. In practice, many sample groups for physiological studies are larger, this arises due to random differences in group size (when a cohort of animals reaches the right age we typically perform analyses on all animals available at that time point to avoid potential bias from selecting only certain animals from the cage/cohort for evaluation). For mostly aged animals, group sizes are often smaller simply for practical reasons (animals are harvested early for tissue collection for example), but we still aim to have group sizes of at least 5-10. In this study, since it was difficult to predict the differences between group means and the within-group variance, we erred toward slightly larger sample sizes. For structural studies (e.g. retinal layer thickness, cone count, etc.) on inbred genetically modified mouse models of retinal degeneration, we have historically observed much less between-animal variation than with functional studies, and sample sizes of 3-5 have been sufficient to detect a difference (e.g. reduction in retinal thickness) of ~20-25%, so that was what we used for this study.
Data exclusions	No data was excluded.
Replication	At least three replicates were made. For quantitative experiments a bigger sample size was selected where possible. Sample sizes are stated in submitted study for each experiment. The observation stated in the study were reproducible.
Randomization	This study does not include treatments so there is no randomization of animals into treatment vs. control groups. Instead the comparisons are between WT and Ush2adelG/delG animals, and as a result assignment to the control (WT) or experimental (Ush2adelG/delG) was made based on genotype. Age was another experimental variable, so was controlled for directly (i.e. control and experimental groups were age-matched for experiments). Animals were raised under the same lighting, housing, and feeding conditions, and were on the same genetic background. Both sexes of animals were used in each experiment and we did not observe any difference in the distribution of males/female in each genotype. In our experience over the past several decades the most important co-variates (i.e. apart from specific mutations which are the experimental variable) affecting retinal degeneration, retinal function, and other retinal phenotypes are age, lighting conditions, and genetic background. All three of those are controlled for in this study by having all animals raised in the same lighting conditions, by having all animals

on the same genetic background (descriptions of breeding are provided in the methods), and by using age-matched groups for all experiments.

Blinding

For all functional (i.e. ERG) and structural (i.e. histology, immunofluorescence, associated microscopy, and subsequent quantitative analysis) animals were only identified by tag number, not genotype or age. For ERG studies, all animals ready at a given time point were analyzed together and were unblinded to group/age only after data collection and analysis (e.g. measurement of amplitudes etc.) were completed. For structural studies such as histology, immunofluorescence, microscopy, and subsequent quantitative analysis (counting cells, measuring layer thickness etc.) where it was important that each experiment contain representatives of each group, one lab member selected tissue sections based on the group (i.e. to ensure that both groups were in the experiment), and then gave slides identified only by tag number to a second lab member who performed the labeling/imaging/analysis. Only after all this was completed were the tag numbers unblinded for statistical analysis and assembly of figures. The only time unblinding (to group) was done prior to the final analysis was for western blots where samples were extracted and protein concentrations measured while identified only by tag number but groups were unblinded prior to loading on the gel so samples could be loaded in a logical order.

Reporting for specific materials, systems and methods

We require information from authors about some types of materials, experimental systems and methods used in many studies. Here, indicate whether each material, system or method listed is relevant to your study. If you are not sure if a list item applies to your research, read the appropriate section before selecting a response.

Materials & experimental systems

n/a	Involved in the study	
☐	☒	Antibodies
☒	☐	Eukaryotic cell lines
☒	☐	Palaeontology and archaeology
☐	☒	Animals and other organisms
☒	☐	Clinical data
☒	☐	Dual use research of concern

Methods

n/a	Involved in the study	
☒	☐	ChIP-seq
☒	☐	Flow cytometry
☒	☐	MRI-based neuroimaging

Antibodies

Antibodies used

Secondary Antibodies:
Alexa Fluor-488, donkey-anti-mouse, Catalog number: A21202, Lot number: 2090565, Supplier: Invitrogen
Alexa Fluor-555, donkey-anti-mouse, Catalog number: A31570, Lot number: 2273774, Supplier: Invitrogen
Alexa Fluor-647, donkey-anti-mouse, Catalog number: A32787, Lot number: TJ271040, Supplier: Invitrogen
Alexa Fluor-488, goat-anti-chicken, Catalog number: A11039, Lot number: 1458638, Supplier: Life Technologies
Alexa Fluor-488, donkey-anti-rabbit, Catalog number: A21206, Lot number: 2289872, Supplier: Invitrogen
Alexa Fluor-555, donkey-anti-rabbit, Catalog number: A32794, Lot number: TI271031, Supplier: Invitrogen
Alexa Fluor-647, goat-anti-rabbit, Catalog number: A21245, Lot number: 1981173, Supplier: Invitrogen
Alexa Fluor-594, donkey-anti-rat, Catalog number: A21209, Lot number: 1547508, Supplier: Life Technologies
Goat Anti-Mouse IgG & IgM Antibody, HRP conjugate, Catalog number: AP130P, Lot number: 3253231, Supplier: Sigma-Aldrich
Goat Anti-Rabbit IgG Antibody, HRP conjugate, Catalog number: AP187P, Lot number: 2709676, Supplier: Sigma-Aldrich
Primary Antibodies:
Provided in supplementary table 1

Validation

CDH23: validated in: Zallocchi, M., Sisson, J. H. & Cosgrove, D. Biochemical characterization of native Usher protein complexes from a vesicular subfraction of tracheal epithelial cells. Biochemistry 49, 1236-1247, (2010).

Centrin2: validated in: Giessl, A. et al. Differential expression and interaction with the visual G-protein transducin of centrin isoforms in mammalian photoreceptor cells. The Journal of biological chemistry 279, 51472-51481, (2004).

Clarin 1: validated in: Zallocchi, M. et al. Localization and expression of clarin-1, the Clrn1 gene product, in auditory hair cells and photoreceptors. Hearing research 255, 109-120, (2009).

Harmonin: validated in: Zallocchi, M. et al. EIAV-based retinal gene therapy in the shaker1 mouse model for usher syndrome type 1B: development of UshStat. PloS one 9, e94272, (2014).

IRBP: validated in: Schneider, B. G., Papermaster, D. S., Liou, G. I., Fong, S. L. & Bridges, C. D. Electron microscopic immunocytochemistry of interstitial retinol-binding protein in vertebrate retinas. Invest Ophthalmol Vis Sci 27, 679-688, (1986).

Opsin (bovine): validated in: Tan, E. et al. The relationship between opsin overexpression and photoreceptor degeneration. Invest Ophthalmol Vis Sci 42, 589-600, (2001).

PCDH15: validated in: Zallocchi, M., Sisson, J. H. & Cosgrove, D. Biochemical characterization of native Usher protein complexes from a vesicular subfraction of tracheal epithelial cells. Biochemistry 49, 1236-1247, (2010).

PRPH2: validated in: Ding, X. Q., Stricker, H. M. & Naash, M. I. Role of the second intradiscal loop of peripherin/rds in homo and hetero associations. Biochemistry 44, 4897-4904, (2005).

SANS: validated in: Zallocchi, M. et al. EIAV-based retinal gene therapy in the shaker1 mouse model for usher syndrome type 1B: development of UshStat. PloS one 9, e94272, (2014).

Usherin b: validated in: Zou, J. et al. Individual USH2 proteins make distinct contributions to the ankle link complex during development of the mouse cochlear stereociliary bundle. Hum Mol Genet 24, 6944-6957, (2015).

VLGR1 (rabbit): validated in: Reiners, J. et al. Scaffold protein harmonin (USH1C) provides molecular links between Usher syndrome type 1 and type 2. Hum Mol Genet 14, 3933-3943, (2005).

VLGR1 (chicken) validated in: Zou, J. et al. Individual USH2 proteins make distinct contributions to the ankle link complex during development of the mouse cochlear stereociliary bundle. Hum Mol Genet 24, 6944-6957, (2015).

Human-20 amino acid: Custom antibody produced by Century Biochemicals. Antibody targets the 20 amino acids inserted due to the frame shift caused by the c.2299delG mutation in usherin. The antibody was only used in IHC. Validation for IHC was done in house via using this antibody on retinal sections of WT mice. The antibody did not show any unspecific staining on WT, while successfully labeling the mutant usherin in our knockin model. This validation was performed three times. The result of one experiment including the absence of staining in the WT retina is displayed in supplemental figure 1E.
The commercial antibodies were validate by their respective supplier:

Sigma-Aldrich, murine primary antibodies (acetylated tubulin (T7451), actin (A3854), FLAG M2 (F1804), GFAP (MAB360), used in immuno blots (IB) and immuno fluorescence microscopy (IF))
Validation for IB and IF: IB using WT and samples in which the target proteins are knocked down or knocked out via siRNA and CRISPR/Cas9, respectively. Confirmation of antibody specificity is validated via absence or diminishing of bands in knockout and knockdown samples, respectively. Knockdown and knockoout are performed in tissue culture. ICC of these cells is used to proof decreased or absent antibody labeling in knockdown and knockout cell samples, respectively. This can be combined with exogenous expression of the target protein in cell lines which do not express the target protein endogenously. Transfection of exogenous protein is followed by knockdown/knockout. Overexpression of the target protein and the resulting increase in band intensity (IB) or labeling (IF) is also applied. For IB and additional test in which the mRNA expression level of the target protein obtained via RNA-seq is compared to the band strength in IB is also performed.

Sigma-Aldrich, Chicken primary antibodies (GAPDH (AB2302) used only in IB)
Validation for IB: IB using WT samples and samples in which the target proteins are knocked down or knocked out via siRNA and CRISPR/Cas9, respectively. Confirmation of antibody specificity is validated via absence or diminishing of bands in knockout and knockdown samples, respectively. Knockdown and knockdown are performed in tissue culture. Overexpression of the target protein and resulting an increase in band intensity (IB) is also applied. An additional test in which the mRNA expression level of the target protein obtained via RNA-seq is compared to the band strength.

Invitrogen, Chicken primary antibodies (Calreticulin (PA1-902A) used only in IF)
Validation for IF: The target protein is either knocked down via siRNA or knocked out via CRISPR/Cas9 in tissue culture. ICC of these cells is checked for reduced or absent labeling in the knockdown and knockout scenario, respectively, when compared to the WT.

Invitrogen, Rabbit primary antibodies (GFP (A11122) used in IB and IF)
Validation for IF: The target protein is either knocked down via siRNA or knocked out via CRISPR/Cas9 in tissue culture. ICC of these cells is checked for reduced or absent labeling in the knockdown and knockout scenario, respectively, when compared to the WT. This can be combined with exogenous expression of the target protein in cell lines which do not express the target protein endogenously. Transfection of exogenous protein is followed by knockdown/knockout.
Validation for IB: The target protein is either knocked down via siRNA or knocked out via CRISPR/Cas9 in tissue culture. IB to check for reduced intensity or absence of the band was performed in the knockdown and knockout scenario, respectively. This can be combined with exogenous expression of the target protein in cell lines which do not express the target protein endogenously. Transfection of exogenous protein is followed by knockdown/knockout.

Cell Signaling, Rabbit primary antibodies (FLAG (14793) used in IB)
Validation for IB: Absence and presence of target band in IB using WT and knockout samples was validated. Samples include both, knockout cell lines as well as knockout mouse models to validate antibody specificity. Further, differences in expression using knockdown cells lines and heterozygous knockout mice are compared to WT via IBs. Additionally, the band intensity obtained by IBs is compared with expression levels obtained via antibody independent assays (RNA-seq, available proteomic data), to proof that band intensity and expression level correlate. A multi antibody strategy using immunoprecipitation (IP) with one antibody against the target protein and subsequent detection with another antibody targeting the same protein was used to further validate antibody specificity. Exogenous expression in tissue culture with subsequent IB to detect the exogenously expressed target protein is used validate antibodies for proteins for which no endogenous expression model is available.

Biolegend, Rat primary antibody (FLAG (637301 used in IF)
Validation for IF: The target protein is either knocked down via siRNA or knocked out via CRISPR/Cas9 in tissue culture. ICC of these cells is checked for reduced or absent labeling in the knockdown and knockout scenario, respectively, when compared to the WT. This can be combined with exogenous expression of the target protein in cell lines which do not express the target protein endogenously. Transfection of exogenous protein is followed by knockdown/knockout.

Proteintech, murine primary antibodies (GAPDH (60004-1-Ig) used in IB)
Validation for IB: The target protein is knocked out via siRNA in tissue culture. IB to validate absence of band in the knockout

scenario.

Proteintech, Rabbit primary antibodies (WHRN (25881-1-AP) used in IF)
Validation for IF: The target protein is knocked out via siRNA in tissue culture. ICC is performed to check for absence of labeling in the knockout cells.

Novus, Rabbit primary antibodies (MWL-Opsin (NB110-74730), Stx3 (NBP1-86984) used for IF)
Validation for IF: The target protein is either knocked down via siRNA or knocked out via CRISPR/Cas9 in tissue culture. ICC of these cells is checked for reduced or absent labeling in the knockdown and knockout scenario, respectively, when compared to the WT. Independent antibodies targeting a different epitope of the same target protein are compared for their localization in cells and tissue. Additionally, a fluorescently tagged version of the target protein is expressed in cells and the localization of the tag is compared to the labeling of the antibody via IF. IF of multiple cell lines, some of which expressing the target protein while other do not express it, are compared to provide an additional level of validation for the antibody specificity.

Santa Cruz Biotech, Goat primary antibody (S-Opsin (sc-14363) used for IF)
Validation for IF: No information provided by Santa Cruz Biotech. We validated the S-Opsin antibody on immunoblots using extracts from wilt type mouse retinas, the cone-like cell line 661W and HEK293 cells. Results using this antibody showed a specific band at the molecular weight of S-Opsin (37.5 kDa) in tested samples of the 661W cell line and mouse retinas but not in HEK extracts. Further validation was performed by using this antibody in immunohistochemical analysis on sections of wild type mouse retinas. The results showed that this antibody identifies cone cells since the labeling was within structures that were also highlighted by labeling with peanut agglutinin, a marker for cone sheaths.

Animals and other research organisms

Policy information about studies involving animals; ARRIVE guidelines recommended for reporting animal research, and Sex and Gender in Research

Laboratory animals	Species: Mus Musculus; Strain: C57BL/6J; Age: P1-P500, specific ages for each experiment are stated in submitted manuscript. Mice were kept at an ambient temperature of 22 C and a humidity of 50%.
Wild animals	No wild animals were used in the present study.
Reporting on sex	Initial experiments showed no sex specific retinal phenotype. Thus, for the experiments described in the submitted sex was not considered and male and female animals were used. No data was collected counting the amount of female vs. male animals used for each experiment. Instead only the total number of used animals is stated.
Field-collected samples	No field-collected samples were used in the present study.
Ethics oversight	All handling, maintenance, and experimental use of animals followed protocols approved by the University of Houston's Institutional Animal Care and Use Committees and were performed according to the NIH and the Association for Research in Vision and Ophthalmology (ARVO) guidelines. Protocol: PROTO201800045.

Note that full information on the approval of the study protocol must also be provided in the manuscript.
