# Supplementary material for: The usherin mutation c.2299delG leads to its mislocalization and disrupts interactions with whirlin and VLGR1
Source: Nat Commun. 2023 Feb 21;14:972. doi: 10.1038/s41467-023-36431-1 (PMC9944904; doi:10.1038/s41467-023-36431-1)

Figure 1B

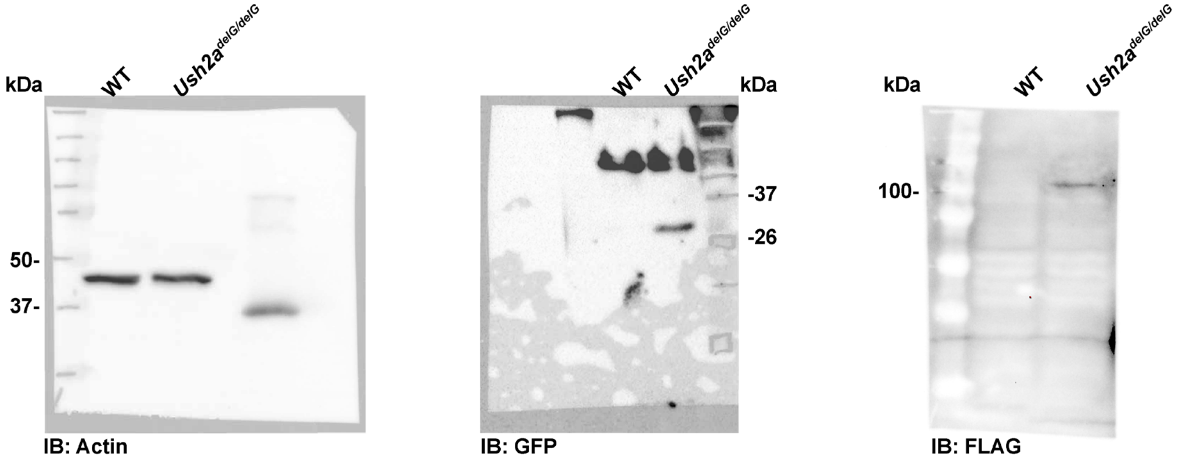

Figure 1C

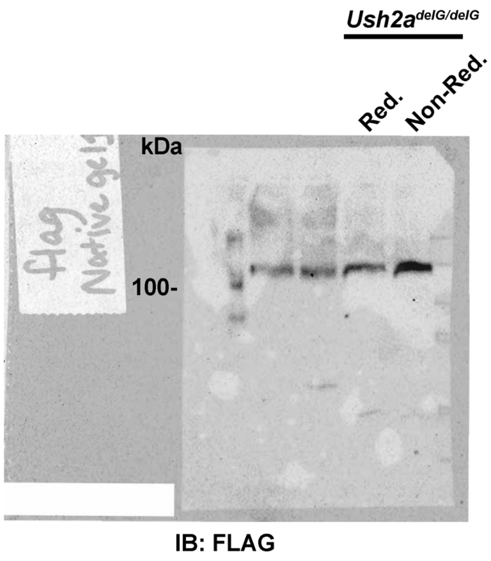

Figure 1D

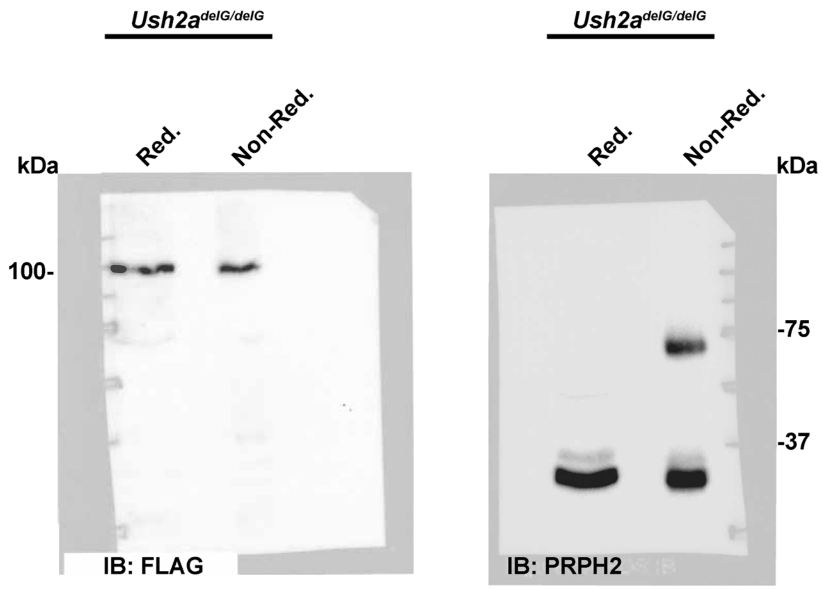

Ush2a delG/delG
Red.
Non-Red.
kDa
100-
IB: FLAG
Ush2a delG/delG
Red.
Non-Red.
kDa
-75
-37
IB: PRPH2

Figure 1I

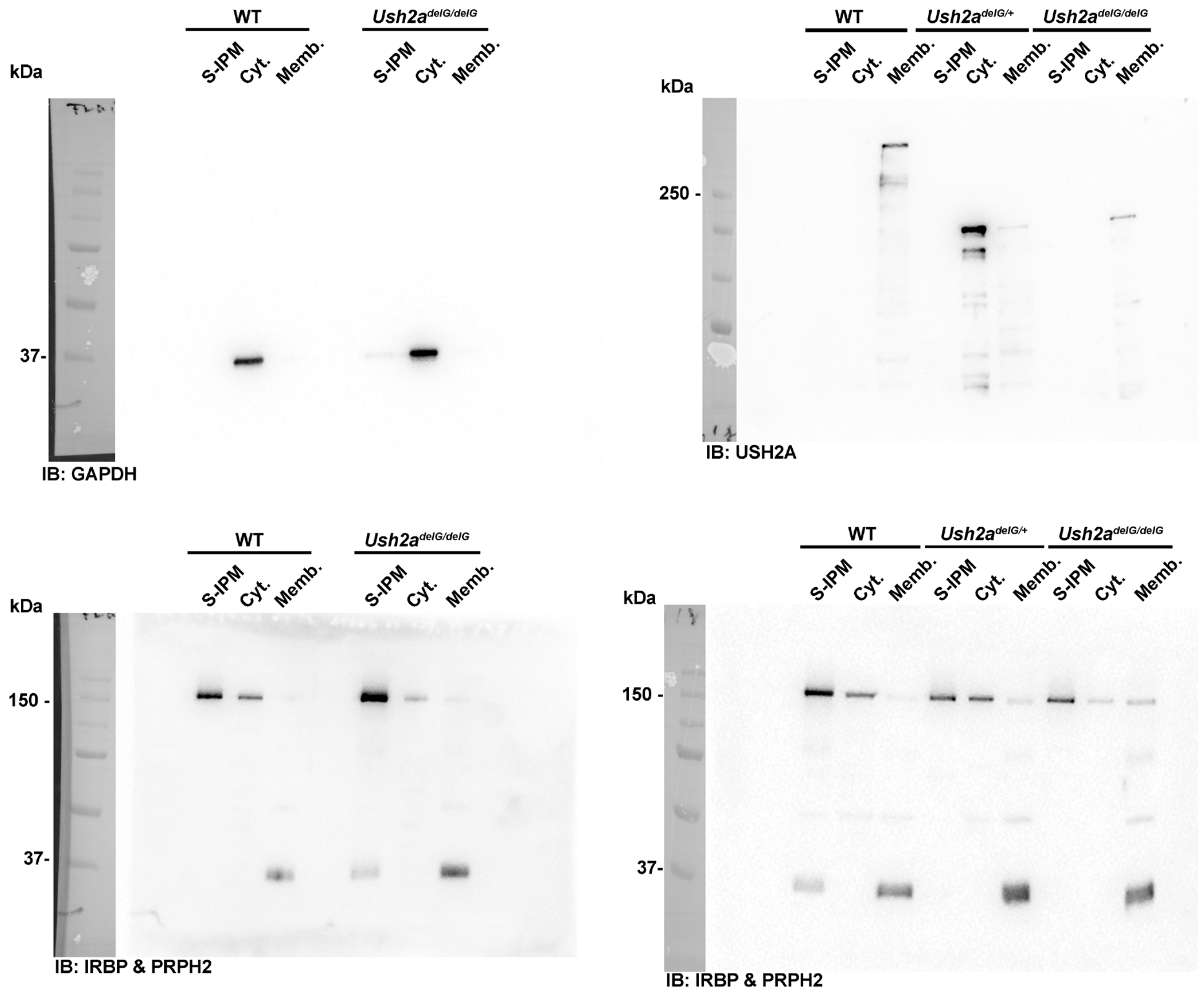

Samples of the same fractionation experiment were loaded on separate Gels. Samples loaded to probe for markers (IRBP, PRPH2, GAPDH) were 1:10 diluted compared to samples loaded to probe for USH2A.

Figure 1J

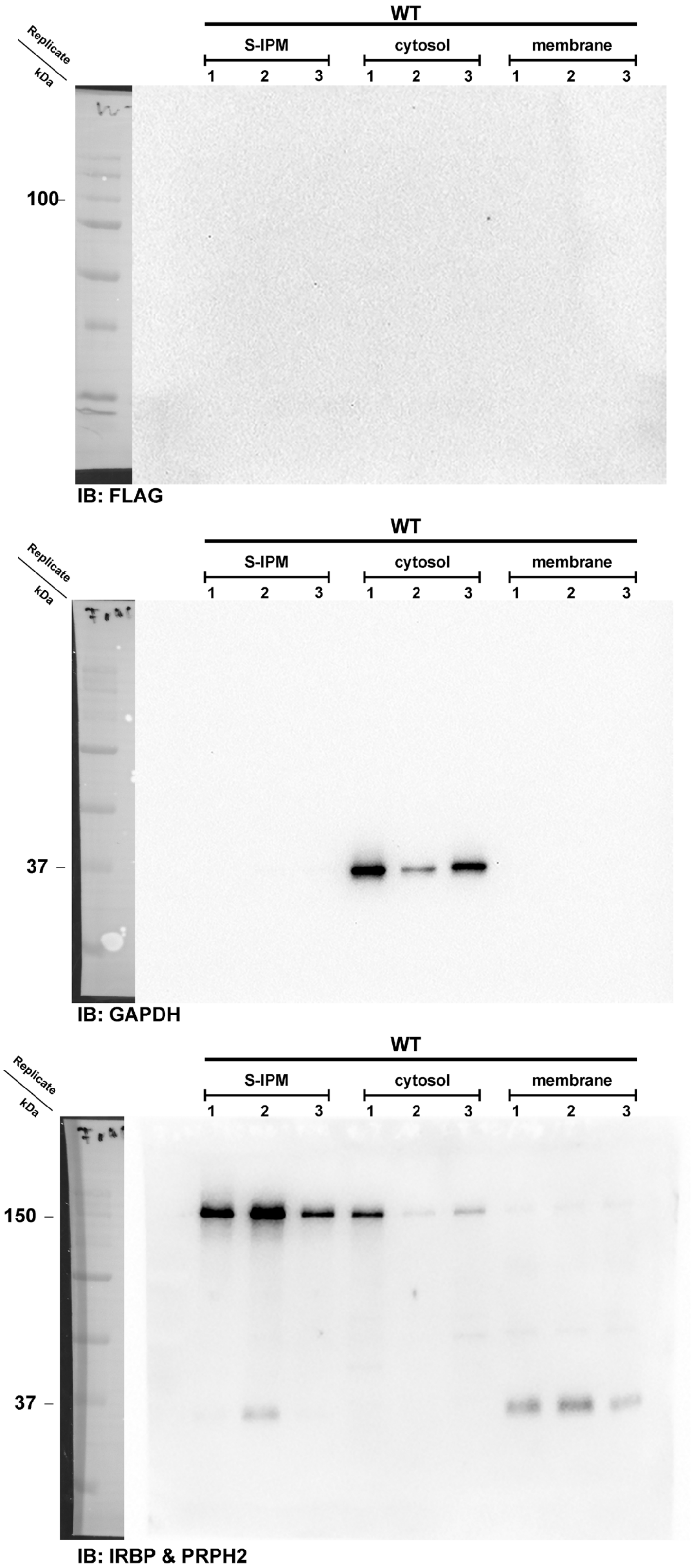

Samples of the same fractionation experiment were loaded on separate Gels. Samples loaded to probe for markers (IRBP, PRPH2, GAPDH) were 1:10 diluted compared to samples loaded to probe for FLAG.

Figure 1K

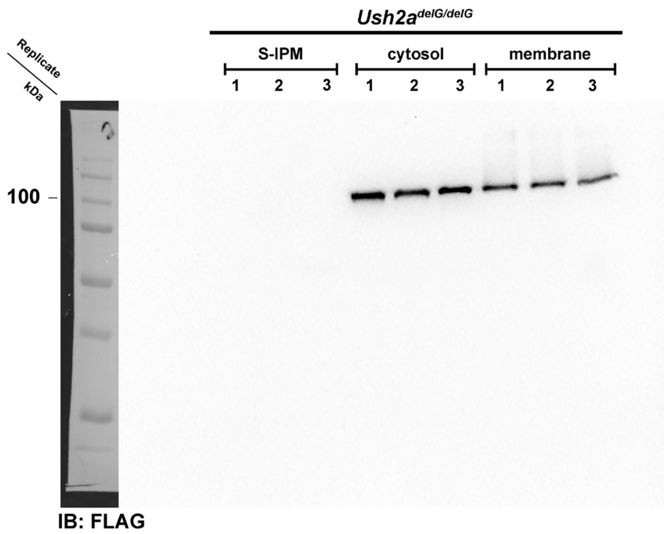

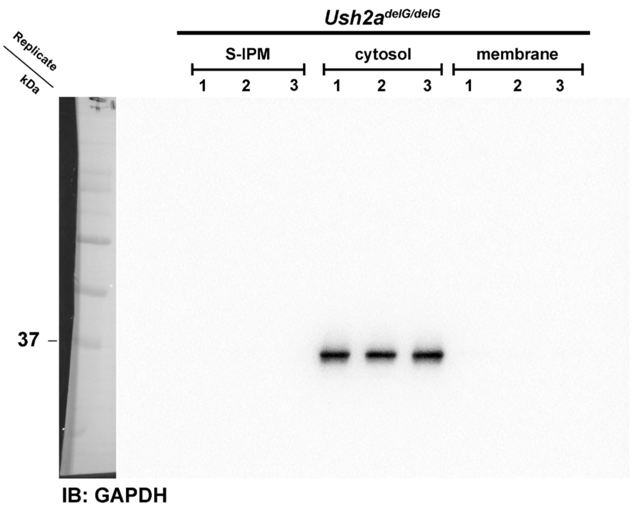

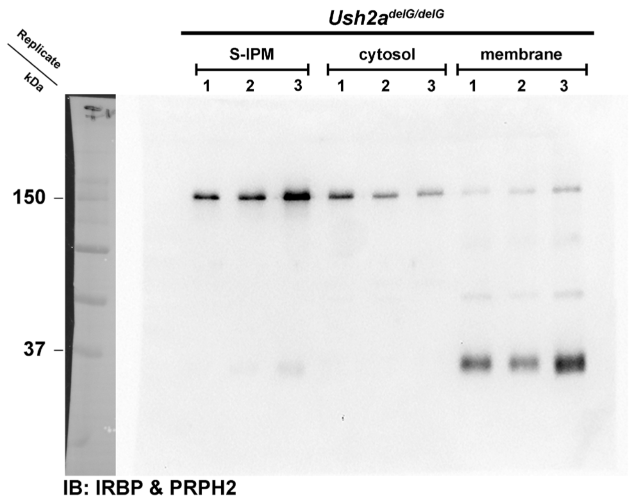

Samples of the same fractionation experiment were loaded on separate Gels. Samples loaded to probe for markers (IRBP, PRPH2, GAPDH) were 1:10 diluted compared to samples loaded to probe for FLAG.

Figure 1L

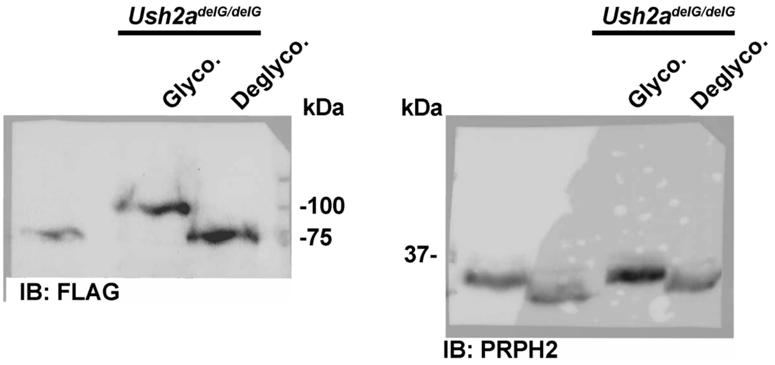
Ush2a delG/delG
Glyco.
Deglyco.
kDa
-100
-75
IB: FLAG
Ush2a delG/delG
Glyco.
Deglyco.
kDa
37-
IB: PRPH2

Figure 8J

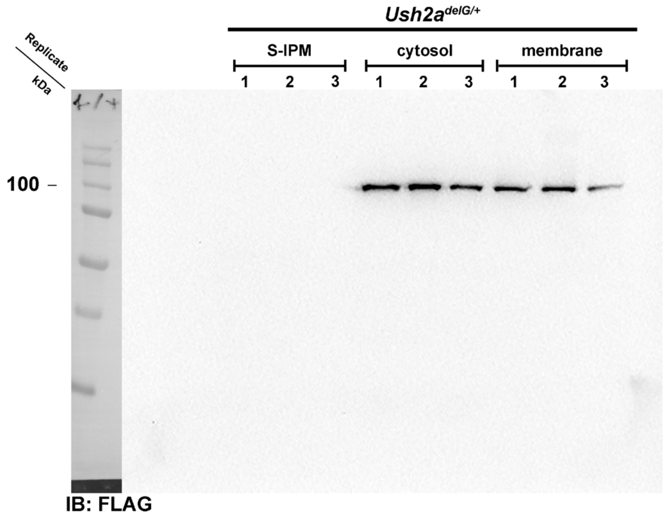

Samples of the same fractionation experiment were loaded on separate Gels. Samples loaded to probe for markers (IRBP, PRPH2, GAPDH) were 1:10 diluted compared to samples loaded to probe for FLAG.

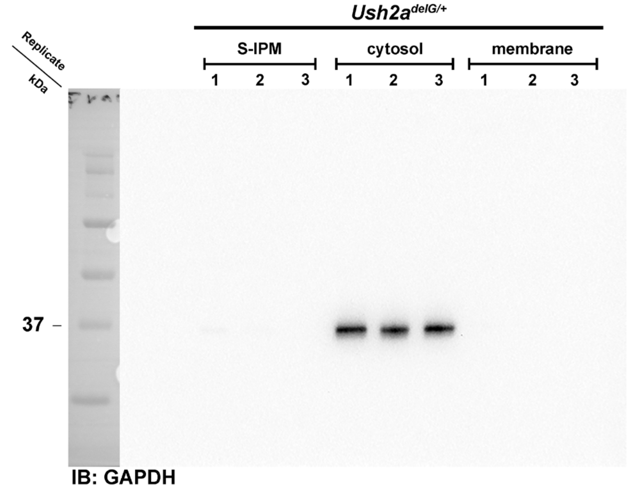

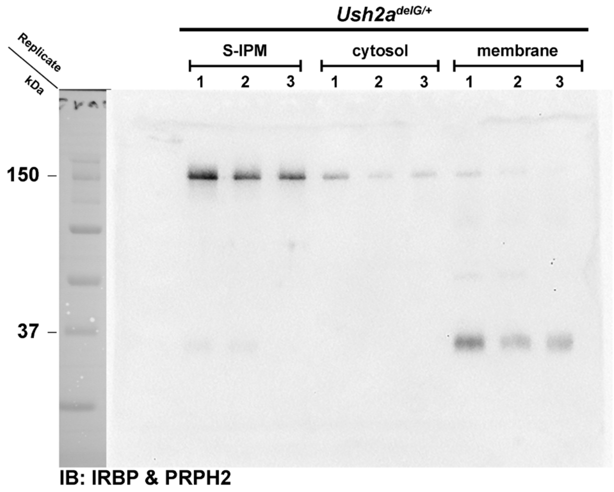

Supplemental Figure 4

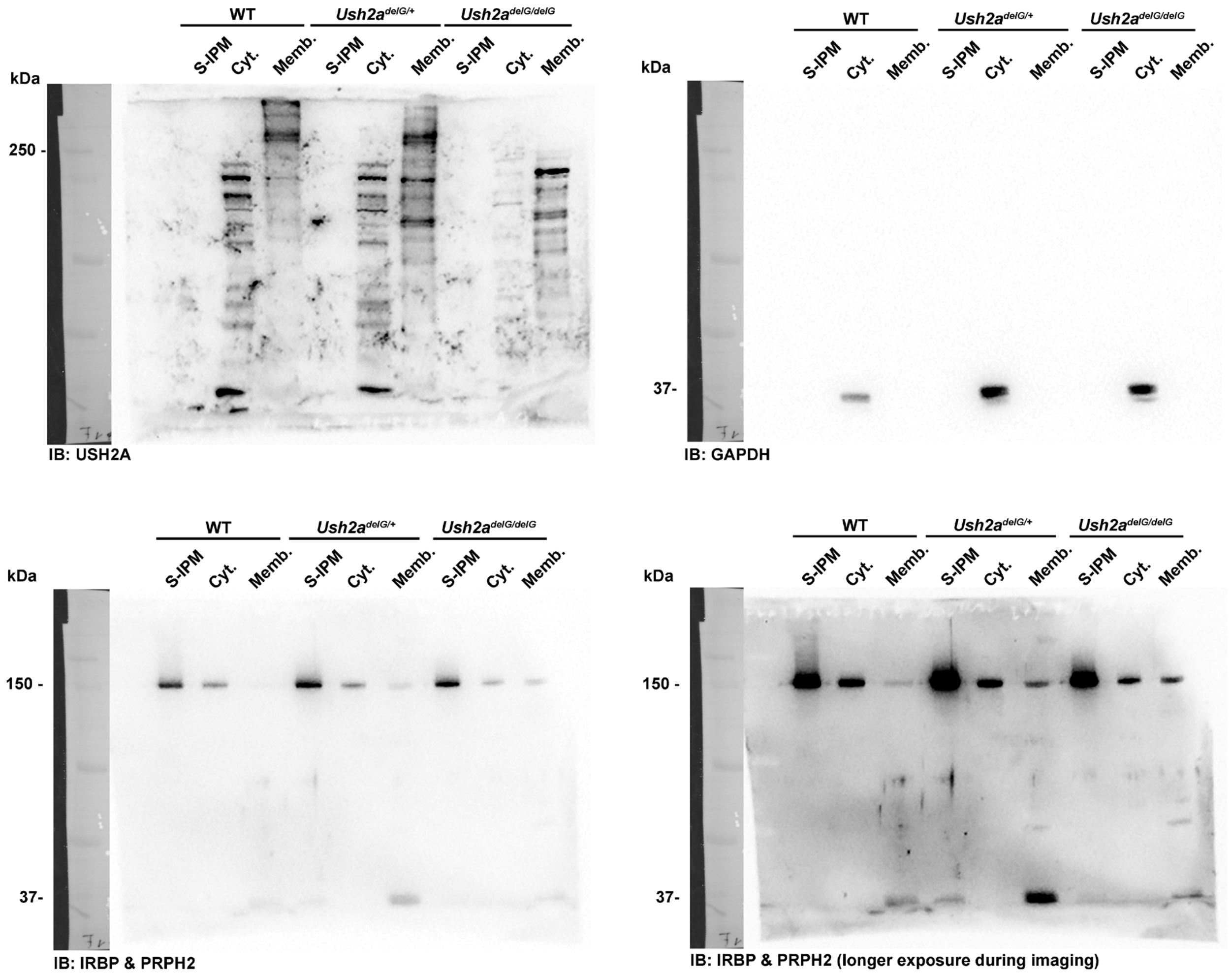

Supplement: Supplementary file 4 — Source Data [file 41467_2023_36431_MOESM4_ESM.zip › Raw blots combined 01-09-23.pdf]
